# Mental Health and Psychological Wellbeing in Rheumatoid Arthritis during COVID-19 – Can Physical Activity Help?

**DOI:** 10.31138/mjr.31.3.284

**Published:** 2020-09-21

**Authors:** Jet J.C.S. Veldhuijzen van Zanten, Sally A.M. Fenton, Sophia Brady, George S. Metsios, Joan L. Duda, George D. Kitas

**Affiliations:** 1 School of Sport, Exercise and Rehabilitation Sciences, University of Birmingham, Birmingham, United Kingdom; 2 Rheumatology Department, Dudley Group NHS Foundation Trust, Dudley, United Kingdom; 3 Faculty of Education, Health and Wellbeing, University of Wolverhampton, United Kingdom

**Keywords:** Rheumatoid arthritis, COVID-19, mental health, psychological wellbeing, physical activity, sedentary behaviour

## Abstract

In response to the COVID-19 pandemic, many countries have adopted community containment to manage COVID-19. These measures to reduce human contact, such as social distancing, are deemed necessary to contain the spread of the virus and protect those at increased risk of developing complications following infection with COVID-19. People with rheumatoid arthritis (RA) are advised to adhere to even more stringent restrictions compared to the general population, and avoid any social contact with people outside their household. This social isolation combined with the anxiety and stress associated with the pandemic, is likely to particularly have an impact on mental health and psychological wellbeing in people with RA. Increasing physical activity and reducing sedentary behaviour can improve mental health and psychological wellbeing in RA. However, COVID-19 restrictions make it more difficult for people with RA to be physically active and facilitate a more sedentary lifestyle. Therefore, guidance is necessary for people with RA to adopt a healthy lifestyle within the constraints of COVID-19 restrictions to support their mental health and psychological wellbeing during and after the COVID-19 pandemic.

On January 30 2020, the World Health Organisation (WHO) declared the coronavirus (now known as COVID-19) a public health emergency of international concern. On March 11, 2020, the Director General of the WHO characterised COVID-19 as a pandemic, due to its ‘alarming levels of spread and severity’. COVID-19 cases and fatalities have now been reported in each continent. Countries have put restrictions in place to prevent the virus from spreading, with the majority of these measures focusing on reducing human contact (eg, via social distancing). These measures are taken to protect healthcare services from being overloaded by increased demand and to protect those who are considered to be at increased risk of developing complications following infection with COVID-19. This includes older adults and people with underlying health conditions, such as Rheumatoid Arthritis (RA) and cardiovascular disease.

The majority of people with RA are taking immunosuppressive medication, putting them at increased risk for infections and comprising their immune response to the virus. In addition, a large proportion of people living with RA also have cardiovascular disease or hypertension,^1^ which by itself are associated with an increased mortality risk of COVID-19.^2,3^ Despite some preliminary evidence that infection risk in RA was not increased during previous coronavirus outbreaks^4^ and that the risk of complications following COVID-19 infections is not increased in people with RA,^5^ healthcare professionals’ recommendations for people with RA on immunosuppressive medication is to self-isolate or shield. These recommendations aim to reduce human interactions, and, therefore, the risk of infection of people with RA. Therefore, it is recommended that this population does not leave their house and mini-mise all non-essential contact with other members of their household during the pandemic. While such measures are deemed necessary to contain the virus and reduce the risk of infection in vulnerable groups such as RA, the potential impact of this social isolation on mental health and psychological well-being should be recognised.

From previous epidemics and pandemics, it is known that isolation and quarantine can impact on psychological wellbeing.^6,7^ Psychological distress, emotional disturbances, irritability, insomnia, post-traumatic stress symptoms have all been reported during or following isolation or quarantine, and these symptoms can last for several months following the end of an epidemic.^6^ The majority of these studies have looked at the impact on wellbeing in individuals who were placed in either isolation or quarantine, due to the development of symptoms (isolation) or exposure to people who might be carrying the virus (quarantine). Given that the virus in previous pandemics seemed to have been contained to specific areas, such as a hospital or hotel (SARS) or specific villages (Ebola), these more localised measures were sufficient to manage the outbreak of these viruses. The public health management of the COVID-19 outbreak is different. On top of these isolation and quarantine measures used for those with symptoms or those who (might) have been exposed to people carrying the virus, community containment is in place affecting whole cities, regions or indeed entire countries to stop the spread of the virus.^8^ Efforts to contain the virus include measures such as social distancing, use of face masks in public places, school closures, and even country-wide “lockdowns”, which are preventing people from leaving their homes, other than for basic necessities (ie, getting food and medication) and critical occupations (eg, health care professionals), and in some countries, to exercise. These measures may lead to compromised mental health and poor psychological wellbeing, through inducing feelings of social isolation, stress (eg, job insecurity, caring responsibilities vs. work commitments), and lack of autonomy (eg, limited access to food and opportunities for physical activity).^9^ For individuals living with RA, it is possible that the added recommendation to shield or self-isolate for a prolonged period of time, coupled with potential concerns about increased infection risk, may magnify such feelings, and significantly negatively impact mental health and psychological wellbeing among these individuals.

Indeed, a survey conducted among 530 people with rheumatic disease (61% RA) during the early days of the current pandemic in the US reported feelings of anxiety, stress, worry, and fear related to COVID-19.^10^ Most of the participants reported themselves to be self-isolating and adopting social distancing measures. Participants expressed worries about their risk of infection and aggravation of their rheumatic disease. Almost half of the participants reported changes in access to health care; some appointments were carried out via teleconferencing, but more frequently appointments were cancelled or postponed, causing frustration. There were also concerns about availability of medication, as hydroxychloroquine has been suggested as a potential treatment for COVID-19, and indeed some patients had experienced difficulties in obtaining their medication.^10^ Even though this information is based on a single study during the start of the pandemic, these findings strongly suggest a negative impact on mental health and psychological wellbeing in RA due to the COVID-19 pandemic. Therefore, appropriate guidance is essential to support people with RA to maintain their mental health and psychological well-being.

Behaviours such as physical activity and engagement in sedentary behaviours (ie, activities requiring ≤1.5 metabolic equivalents, undertaken in a sitting or reclining posture^11^) can impact on wellbeing. In RA, physical activity has been shown to induce many benefits in regard to both physical and psychological health,^12–16^ whereas sedentary behaviour is related to poorer health outcomes in this population.^17–19^ Several physiological and psychological mechanisms have been suggested through which physical activity can improve indices of psychological health. Examples of physiological mechanisms are a physical activity-induced decrease in systemic inflammation and increase brain derived neurotrophic factor (BDNF) and brain-derived monoamines, which have all been associated with better mental health.^20^ Examples of psychological mechanisms through which physical activity can improve mental health and psychological wellbeing include increases in general feelings of self-efficacy and self-esteem, distraction from stress and worries, as well as physical activity-induced positive affect.^20–23^ It is therefore not surprising that the WHO has recommended exercise and physical activity as a way to cope with stress during COVID-19,^24^ and several countries have included engagement in daily physical activity as one of the few permitted reasons to leave the house. During the SARS outbreak in Hong Kong, people who were more worried or apprehensive due to SARS and experienced more work or finance-related stress, reported to have increased their levels of exercise,^25,26^ suggesting that exercise was used as a mechanism to cope with the stress, and perhaps it was used as a way for individuals to take control of their health.^27^ In contrast, those who felt helpless due to SARS were more likely to report lower levels of exercise during the epidemic.^25,26^ Taking together, this research suggests that exercise can indeed be used to cope with stress evoked during an epidemic, but behaviour change support which is tailored to personal circumstances is necessary when encouraging people to be more active and less sedentary.

To stay or become physically active during a pandemic is difficult, and emerging evidence based on self-report data and activity trackers shows lower levels of physical activity during COVID-19 lockdown periods in the general population.^28–30^ Being physically active during COVID-19 is even more difficult for people with RA who often experience RA-related barriers to physical activity.^31^ For example, closures of fitness centres limits access to exercise equipment and fitness classes tailored to people with RA. People with RA who are able to continue to work at home while shielding have no longer the opportunity for active commuting. Desks/workstations at home are often less adapted to individualised ergonomic needs (eg, no access to sit/stand desk when working on the dining room table). Those who are not able to work from home, which are often the more active occupations, are more likely to sit at home, meaning they have exchanged their occupational physical activity for more sitting. These are all different examples on how the current pandemic can encourage less physical activity and more sedentary behaviour in RA, a population that is already characterised by lower levels of physical activity and higher levels of sedentary behaviour.^17,18,32^

Since the start of the pandemic, there has been a plethora of advice and recommendations readily available in the news and online, suggesting ways in which people can stay physically active during the pandemic and imposed “lockdown” restrictions. For example, social media has seen an exponential growth in recommendations and suggestions for home-based exercise, which can be carried out during the lockdown. However, due to RA-related barriers to physical activity,^31^ these generic exercise programmes are not suitable for widespread use among this population. Thus, more tailored guidance is necessary for people living with RA. In order to provide appropriate behavioural support to be more physically active and less sedentary, it is important to first explore the determinants of these behaviours. Currently, we know very little about how the pandemic has impacted on physical activity and sedentary behaviours in RA, or any other clinical population. So far, data suggest that some people with rheumatic disease have reported difficulties in adapting their physical activity to social distancing requirements as they commonly exercised indoors with friends, whereas some have changed their activities to engage in more walking or running.^10^ More research is necessary to explore the impact of the pandemic on physical activity and sedentary behaviours and its association with mental health and psychological wellbeing in RA. We need more evidence to ascertain if any modifications in behaviour (and potential impacts on mental health and well-being) take place either during or after the pandemic restrictions ease. This will provide essential information on how people living with RA can be supported to maintain a healthy lifestyle while experiencing restrictions related to the pandemic, as well as during the aftermath of the pandemic when the negative impacts on psychological health and well-being may continue.^6^

As a final note, it is important to emphasise that improvements in mental health and psychological wellbeing can be achieved by relatively small changes in behaviour (eg, increasing levels of physical activity and/or decreasing time spent sedentary). Awareness of this may facilitate perceptions among people living with RA that behaviour change in this domain is much more feasible. While physical activity guidelines recommend 150 minutes of moderate-intensity physical activity each week – including for people with RA – there is no lower threshold at which the benefits of physical activity are evident. The recently revised UK physical activity guidelines now include the messages of ‘every minute counts’ and ‘some is good, more is better’. Indeed, light-intensity physical activity (such as standing, slow walking, and lifestyle-embedded activities of daily living) is related to lower risk of depression and better psychological wellbeing,^33^ as well as lower mortality rates.^34,35^ Similarly, modest changes in sedentary behaviour are also reported to be beneficial for mental health and psychological wellbeing. For example, replacing 30 minutes of sedentary behaviour with light-intensity physical activity during a day has been suggested to reduce anxiety and improve psychological wellbeing.^36^ Therefore, it is recommended that these messages like ‘every minute counts’ and ‘some is good, more is better’ are emphasised when advising people with RA to be more active and less sedentary to support their mental health and psychological wellbeing during the pandemic.
